# Ileocolonic Intussusception Secondary to Colon Cancer: A Rare Cause of Abdominal Pain In Adults

**DOI:** 10.7759/cureus.64442

**Published:** 2024-07-13

**Authors:** Mohamed Ahmed, Ahmed Allawi, Naofal K Da Silva, Rasha Saeed, Danya Auda

**Affiliations:** 1 Surgery, University of California, Riverside, USA; 2 Surgery, AdventHealth Tampa, Tampa, USA; 3 Occupational Medicine/Environmental Medicine, University of California, Irvine, Irvine, USA; 4 Psychology, University of California, Riverside, USA

**Keywords:** computerized tomography, robotic surgical procedures, non-bloody diarrhea, unexplained abdominal pain, ileocolonic intussusception

## Abstract

Intussusception, defined as the telescoping of one segment of the gastrointestinal tract into an adjacent one, is a rare cause of abdominal pain in the adult population due to underlying benign or malignant pathology. With the liberal use of CT in the evaluation of patients with abdominal pain, the diagnosis became more reliable. Resection of the bowel segment is the recommended treatment in most cases. We are presenting the case of a 76-year-old male patient who presented with a three-week history of abdominal pain and diarrhea. The evaluation was consistent with ileocolic intussusception. Robotic resection of the right colon was performed. Pathology revealed poorly differentiated adenocarcinoma of the cecum as the underlying pathology.

## Introduction

Intussusception is defined as the invagination of a proximal bowel segment into the lumen of an adjacent distal segment. The incidence is two to three cases per 1,000,000 per annum. The age of presentation is highly variable, ranging from the neonatal period to the seventh decade of life [1]. Adult intussusception accounts for approximately 1% of all bowel obstructions and 5% of all intussusceptions with an incidence of 0.003-0.02% for in-hospital admissions [2]. The clinical presentation is often nonspecific, with symptoms and signs of bowel obstruction being common including but not limited to continuous abdominal pain, nausea with intermittent some sometimes feculent vomiting, variable degree of abdominal distention, and diffuse abdominal tenderness. Typical colicky abdominal pain is a common presentation in the pediatric population but is less common in adults [3]. Given this variability in presentation, the preoperative diagnosis can be challenging. Abdominal imaging, specifically CT scan, is the most effective tool with high sensitivity and specificity [4]. The etiology of adult intussusception can be due to benign, malignant, or idiopathic pathology [5].

## Case presentation

**Figure 1:**
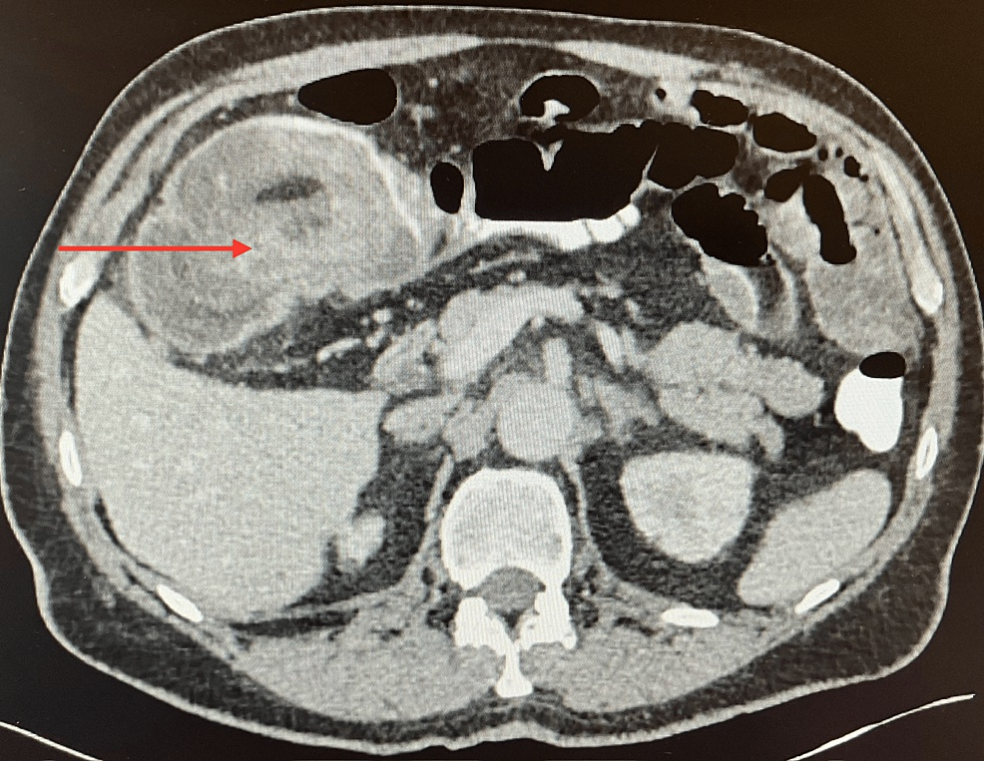
CT abdomen (axial) showing right colon intussusception (red arrow)

A 76-year-old otherwise healthy male presented to the emergency room with a three-week history of colicky abdominal pain and watery diarrhea. The patient denied any family history of colon-rectal cancer nor had a colonoscopy in the past. Laboratory findings revealed a normal white blood cell count of 9.44x10^3^/ul (reference range, 4.8-10.8x10^3^/ul), hemoglobin 13 g/dl (reference range, 14.0-18.8 g/dl), blood urea nitrogen (BUN) 17 mg/dl (reference range, 7.0-18.0 mg/dl), creatinine 0.9 mg/dl (reference range, 0.60-1.3 mg/dl). The patient was hemodynamically stable, and afebrile, and physical evaluation revealed a non-distended soft abdomen with no peritoneal signs. A CT scan showed a long segmental ileocolic intussusception involving the right colon extending the proximal transverse colon with a mass (leading lesion) concerning underlying malignancy (Figure 1).

**Figure 2:**
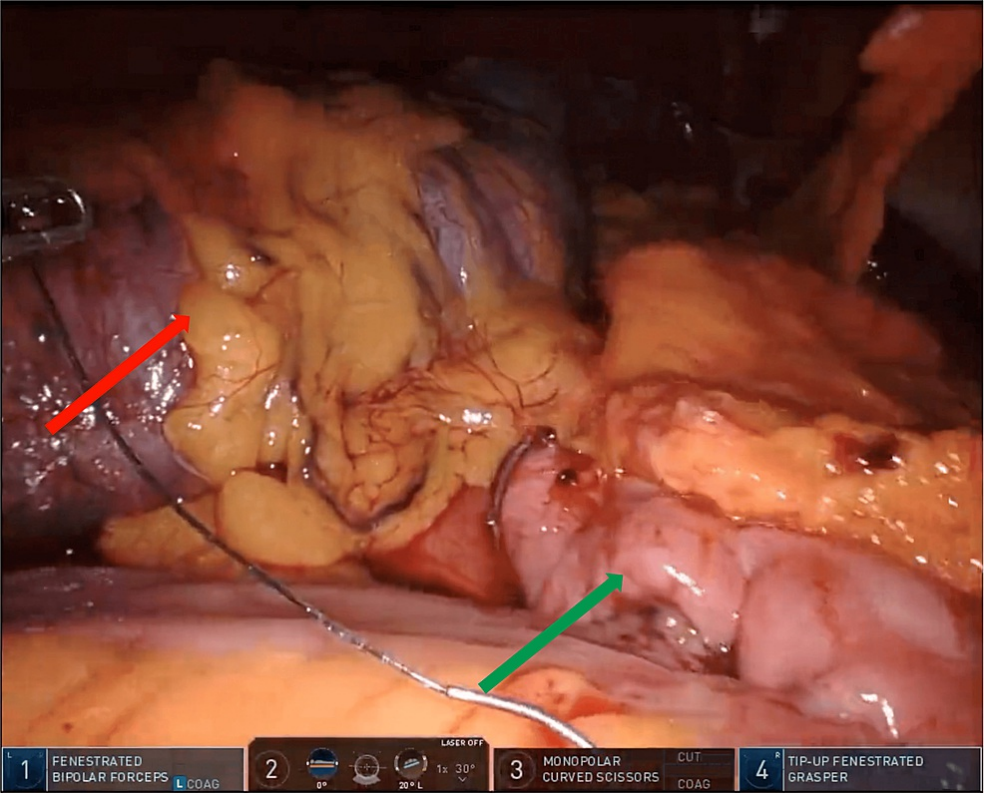
Robotic resection of the right colon Resected right colon (red arrow), Ileocolic anastomosis (green arrow)

The patient was taken to the operating room and robotic right colon resection with primary anastomosis was performed (Figure 2).

The operative challenge was performing a high vascular ligation (ileocolic and right branch of the middle colic) as the mesentery of the ascending colon was also intussuscepting (Video 1).

**Video 1:** Robotic right hemicolectomy in a case of ileocolic intussusception

The patient had a non-eventful postoperative recovery and a regular oral diet was started on postoperative day 1. He was discharged from the hospital on postoperative day three. Pathology revealed intussusception with poorly differentiated invasive adenocarcinoma of the cecum (6.2x6x3 cm), 23 lymph nodes, negative for metastatic carcinoma.

## Discussion

Paul Barbete was the first to describe Intussusception in 1674 as the invagination of the proximal portion of the intestine (intussusceptum) into the distal portion of the intestine (intussuscipiens) in a telescope-like fashion [6]. Sir Jonathan Hutchinson first described a reduction of intussusception in 1871 [7]. Intussusception commonly occurs at the junctions between freely moving segments and retroperitoneally or fixed segments secondary to adhesions [8].

Symptoms include episodes of intermittent abdominal pain and vomiting [9]. Patients may present with bowel obstruction symptoms including crampy abdominal pain (71%), nausea and vomiting (68%), abdominal fullness (45%), and tenderness (60%) [10]. In the adult population, intussusception is the result of a malignant colonic lesion in 43% of patients and benign pathology in 57% of patients with CT scanning as the most useful diagnostic radiologic method, with the typical target or sausage signs being the most common finding [11]. 

Barium enema or colonoscopy can reveal, reduce, and diagnose the cause of intussusception guiding treatment decisions [12]. Surgical resection without reduction is the main treatment for intussusception due to the high probability of malignant etiology. Minimally invasive approaches such as laparoscopic or robotic surgery have been frequently employed given their favorable shorter stay and lower postoperative pain profile. However, when benign pathology is suspected polys or colon lipoma endoscopic reduction and resection of small <2 cm benign tumors can be an option [13,14]. Robotic resection is safe and effective for complex cases in colorectal surgery [15].

## Conclusions

Colon intussusception is rare in adults and can present with vague symptoms. Abdominal pain can be the presentation. CT scanning is the most useful diagnostic radiologic method. Enema and colonoscopy can be helpful and endoscopic resection is recommended for benign tumors less than 2 cm. Surgical oncological resection of the intussusception without reduction is the treatment in the elderly when underlying malignancy is suspected. Robotic resections yield more harvested lymph nodes, finer resection margins, and shorter hospital lengths of stay.
